# Quality by Design (QbD) based process optimisation to develop functionalised particles with modified release properties using novel dry particle coating technique

**DOI:** 10.1371/journal.pone.0206651

**Published:** 2018-11-01

**Authors:** Eman Z. Dahmash, Ali Al-khattawi, Affiong Iyire, Hamad Al-Yami, Thomas J. Dennison, Afzal R. Mohammed

**Affiliations:** Aston School of Pharmacy, Aston University, Birmingham, United Kingdom; University College Cork, IRELAND

## Abstract

Quality by Design (QbD), a current trend employed to develop and optimise various critical pharmaceutical processes, is a systematic approach based on the ethos that quality should be designed into the product itself, not just end tested after manufacture. The present work details a step-wise application of QbD principles to optimise process parameters for production of particles with modified functionalities, using dry particle coating technology. Initial risk assessment identified speed, air pressure, processing time and batch size (independent factors) as having high-to-medium impact on the dry coating process. A design of experiments (DOE) using MODDE software employed a D-optimal design to determine the effect of variations in these factors on identified responses (content uniformity, dissolution rate, particle size and intensity of Fourier transform infrared (FTIR) C = O spectrum). Results showed that batch size had the most significant effect on dissolution rate, particle size and FTIR; with an increase in batch size enhancing dissolution rate, decreasing particle size (depicting absence of coated particles) and increasing the FTIR intensity. While content uniformity was affected by various interaction terms, with speed and batch size having the highest negative effect. Optimal design space for producing functionalised particles with optimal properties required maximum air pressure (40psi), low batch size (6g), speed between 850 to 1500 rpm and processing times between 15 to 60 minutes. The validity and predictive ability of the revised model demonstrated reliability for all experiments. Overall, QbD was demonstrated to provide an expedient and cost effective tool for developing and optimising processes in the pharmaceutical industry.

## Introduction

Quality in the pharmaceutical industry has traditionally been assured via quality by testing (QbT), where failure results in whole batches being discarded at significant cost. In this approach, a lack of understanding of the critical process parameters (CPPs) renders manufacturing issues difficult to diagnose and can lead to substantial losses [1,2]. Alternatively, quality by design (QbD) provides a unique opportunity for the pharmaceutical industry to dramatically enhance product quality via utilising a systematic risk based approach [3]. This can offer significant advantages over QbT via allowing control of the manufacturing process to ensure consistent production of a quality product, without the need of extensive quality testing [4]. The use of QbD is becoming an integral part of the development and optimisation of novel manufacturing processes as the industry and regulatory authorities move together towards an agile system for pharmaceutical product development.

**Fig 1:**
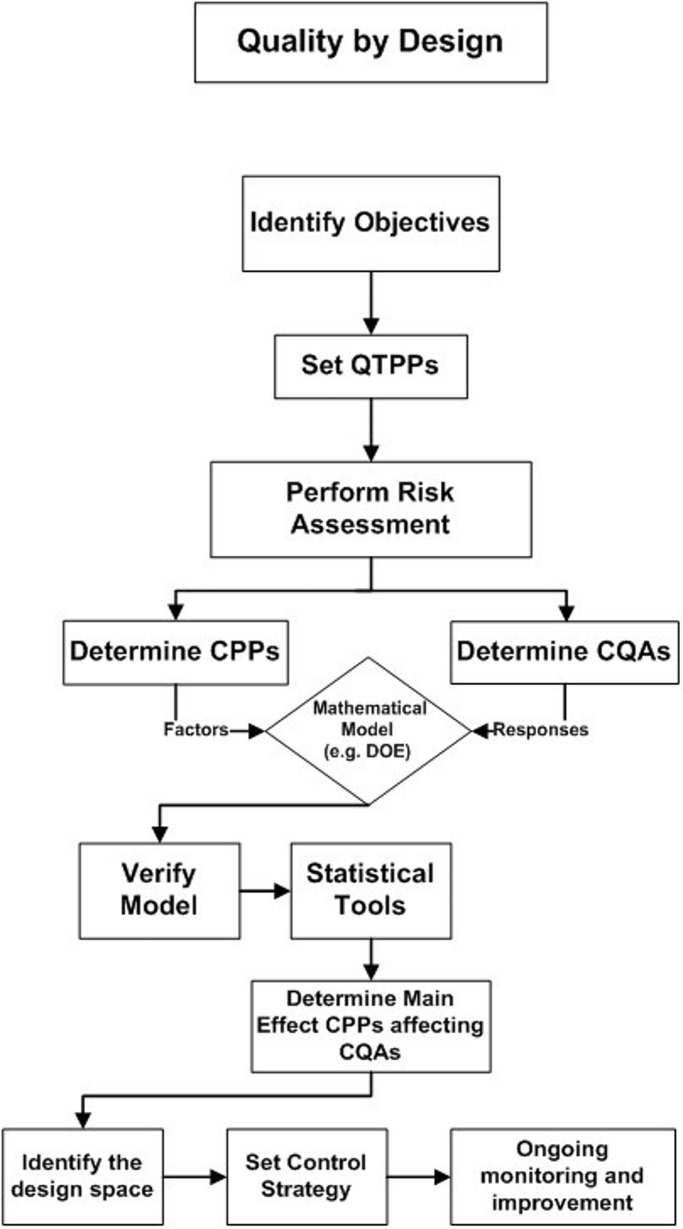
Flow chart outlining the elements of QbD (QTPP: Quality targeted product profile, CPPs: Critical process parameters, CQAs: Critical quality attributes, DOE: Design of experiments).

QbD provides a step-wise approach for process optimisation with elements that once established, will help product features to be recognized and origins of inconsistency identified [5]. An outline of this approach is depicted in Fig 1. Briefly, the quality target product profile (QTPP) must first be defined and inclusive of requirements for quality, safety and efficacy. These form the basis for product design and should be reproduced constantly to deliver the intended benefits [3,6,7]. Subsequently, risk management, as detailed in ICH Q9, is applied to identify CPPs in a product’s manufacturing process that directly impact the product’s critical quality attributes (CQAs). The latter could comprise the physical, chemical, biological or microbiological features/specifications that are required in a suitable limit to achieve desired product quality [7,8]. Using risk-assessment tools, CPPs and CQAs are determined for inclusion and investigation via design of experiment (DOE) studies. DOE is a tool by which systems and processes can be investigated to give an understanding of the main and interaction effects of various CPPs and provide flexibility by predicting the extent of these interactions [9,10].

**Fig 2:**
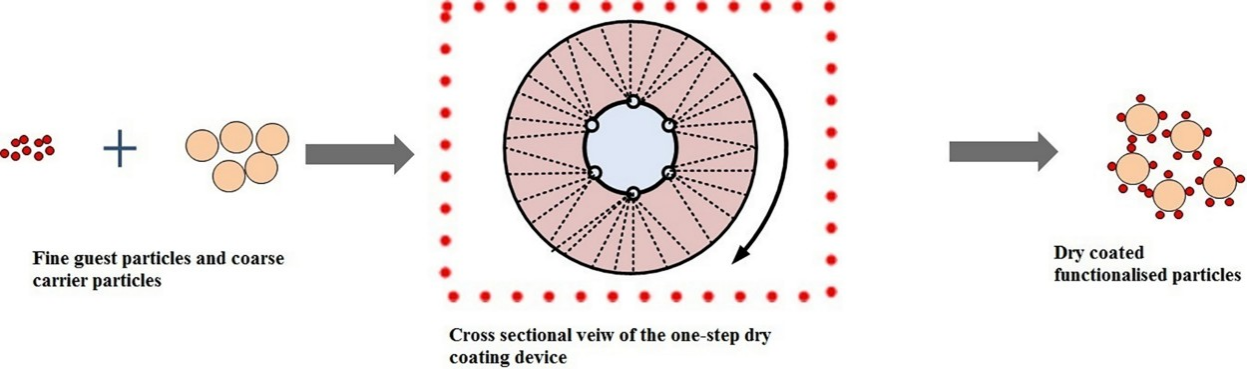
Schematic showing dry coating process and device. Fine agglomerated guest particles and coarse carrier particles are added to the high G-force processing vessel coupled with nitrogen gas flow to produce dry coated FP in a one step process.

A process that benefits from optimisation via a QbD approach is dry particle coating. This novel process enables the production of functionalised modified release particles (FP). It involves fine particles (guest, e.g. drug that requires modification of release kinetics) adherence on to the surface of coarse particles (carrier) through the application of high mechanical and impaction forces without the use of heat or solvents (Fig 2) [11–16]. In the process, the attraction of guest to carrier particles is based on van der Waals forces, electrostatic forces and hydrogen bonding [17,18]. Dry coating requires initial de-agglomeration of the cohesive guest particles into primary particles prior to coating the carrier. The equipment was designed to supply sufficient G-force (force of acceleration of an object) from a rotating drum coupled with high nitrogen gas flow essential to break agglomerates and promote proximity between the guest and the carrier particles. However, the cohesive nature of fine guest particles results in difficulty to disperse them and therefore, obtaining a uniform mix is challenging [19]. A plethora of factors in the dry coating process may contribute to the optimum product performance including blending speed, time, air flow rate and batch size. Elucidating the effect of the individual process variables as well as their interactions, therefore, is crucial to facilitate the development of future products using this technique.

This paper aims to optimise the process parameters of a dry particle coating technology via applying a step-wise QbD approach. Specifically, the work is comprised of screening studies to generate initial understanding of the process variables that influence the operation of the technique and development of functionalised particles with modified release properties. These studies were focused mainly on assessing powder homogeneity/content uniformity, release profile using dissolution studies and the extent of interaction between the guest and carrier particles via particle size analysis and FTIR techniques. Risk assessment was then performed following both compendial and initial screening studies to identify CPPs that have the highest impact on CQAs. Those were subsequently incorporated into an optimisation DOE study based on a four-factor, multi-level quantitative D-optimal design plan. Response surface model (RSM) was applied in this study to evaluate the effect of varying interactive terms among CPPs (processing speed, time, airflow and batch size) on four CQAs (drug release, blend homogeneity and degree of attraction of guest to carrier particles). Finally the model was utilised to establish the design space of the process.

## Materials and methods

### Materials

Ibuprofen was purchased from Discovery Fine Chemicals (Dorset, UK), whereas microcrystalline cellulose (MCC) Avicel PH-200 was donated by FMC BioPolymer Europe (Brussels, Belgium).

### Methods

#### Preparation of FP using dry particle coating process

FP were produced using a dry particle coating process, where a model binary mix comprising of ibuprofen fine guest particles and MCC as coarse model insoluble carrier was chosen. The required particle size ranges were obtained by sieving. MCC particle size was 180–250 μm and ibuprofen particle size was 38–53 μm (collecting the fraction of powder retained above sieve with pore size 38 μm and below sieve with pore size 53 μm). Both the carrier and the guest materials (10% w/w guest concentration, 5g) were added to the dry particle coating device chamber, (prototype developed by the research group at Aston University, UK) as shown in Fig 2. The mixing process parameters in terms of speed, processing time and nitrogen gas pressure for the optimisation study was carried out according the parameters set by the MODDE design worksheet. At the end of each run, resultant powder was collected into a glass container and assayed for dissolution profile, content uniformity, particle size distribution and Fourier Transform Infrared (FTIR). For screening experiments, the resultant composite powders were tested against physical mixtures (control) made of the same components that were mixed manually in a 30mL glass vial with cap for 5 minutes (manual vigorous shaking).

#### Dissolution studies

Dissolution studies were carried out according to the USP apparatus I compendia basket method [20]. Sample size was an equivalent of 100 mg of ibuprofen (1 g of the blend). The amount of the released ibuprofen was determined by UV spectrophotometry set at wavelength of 272 nm (Jenway 6305, Staffordshire, UK). All experiments were performed in triplicates.

#### Content uniformity

Samples of the blend containing an equivalent of 50 mg of ibuprofen were accurately weighed, added to a flask and dissolved in 100 ml of phosphate buffer (pH 7.2). Ibuprofen content was assessed using UV spectrophotometry at 272nm. The degree of homogeneity of the mixture was measured by the relative standard deviation (RSD) using Eq 1 [21].

$$RSD=\frac{\sqrt{\sum_{i=1}^{n}\frac{{\left(x_{i}-\bar{x}\right)}^{2}}{n-1}}}{\bar{x}}$$ Eq (1)

Where *x*_*i*_ *is* the ibuprofen content in percentage in the sample, $\bar{x}$ is the average concentration over all samples and *n* is the number of samples. All samples were made in triplicates.

#### Particle size analysis (PSA)

PSA was carried out using laser diffraction particle size analyser Sympatec HELOS (Helium–Neon Laser Optical System/ RODOS). A3 lenses were employed with working range of 0.5–175 μm. The equipment disperses particles into their primary component using dry disperser (RODOS) with compressed air set at 3 bars. 1 g was placed over the feeding tray of VIBRI, which in turn transferred the sample into RODOS. Parameters like volume mean diameter (VMD) (average diameter of the particles that has the same volume), X_10_, X_50_ and X_90_ were obtained. The X_10_ X_50_ and X_90_ values represent the particle dimension corresponding to 10%, 50% and 90% of the blend in the cumulative undersize distribution respectively. All samples were performed in triplicate.

#### Fourier transform infrared (FTIR)

FTIR spectra were recorded on solid samples using Thermo Scientific Nicolet iS5 FTIR spectrometer (Hertfordshire, UK), coupled with Thermo Scientific OMNIC software (Nicolet, Madison, WI) to operate and treat FTIR spectra. Samples of approximately 10–20 mg were loaded on the iD5 ATR Diamond sample holder and triplicate scans were obtained over the range of 500–4000 cm^-1^ with a resolution of 2 cm^-1^.

#### Statistical analysis

Statistical comparison in screening studies for dissolution profiles for various formulations was done using t-test (two-tails) whereas; comparisons between several profiles were carried out using two-way analysis of variance (ANOVA). All studies were carried out in triplicate and the mean with standard deviation was reported throughout screening experiments.

#### Elements of QbD

CQAs were identified based on the initial screening studies and literature review. Risk assessment was then employed during development to identify possibly high risk input and process parameters to conclude the required variables that would be investigated. Risk ranking was determined based on the screening studies, current knowledge and the ICH Q9 risk management guidelines [8]. The CPPs were assessed against CQAs using risk assessment tool. For each input and process attribute a risk rank was made based on the impact on the final product quality. Identification of CQAs of the final product were determined also according to risk assessment anticipating a failure of the product if CQAs were not met. Risk ranking was categorised as low, medium and high. Risks ranked as low were not included as independent factor, while those with medium and high risk rank were considered as independent factors and used in the DOE study [6,8].

#### Design of experiment (DOE)

DOE was statistically designed using MODDE software version 8.2 (Umetrics Inc., NJ, USA). A D-optimal design was selected to account for the asymmetry in the settings of critical process parameters, namely process speed and airflow. The asymmetric nature of the selected D-optimal design suggests that sometimes reproducibility (i.e. variation of response under the same conditions, pure error) could be tested at levels other than middle values [10]. Hence reproducibility runs were set at the high level values by the software. The chance for error during the model design was minimized through ensuring that both G-efficiency (measure of model efficiency) and condition number (measure of model sphericity) were at their optimal levels of 85.15% (recommended > 60–70%) and 10.55 respectively [22]. The model was quadratic polynomial fitted using partial least squares (PLS) method. Response surface modelling (RSM) was applied to estimate the non-linear multidimensional relationship between the independent factors and CQAs. To fit the quadratic model with D-optimal design, 26 runs were generated of which four of these were replicated to estimate the pure error for the study (Table 1). The randomization of the order of the experiments was carried out. Further, Table 2 summarises the independent factors used in the design.

**Table 1:** The D-optimal design worksheet with CPPs and the total number of runs.

Exp. No	Speed	Time	Air Flow	Batch Size
	**(rpm)**	**(min)**	**(psi)**	**(g)**
1	300	15	0	6
2	300	60	0	6
3	1500	60	0	6
4	1500	15	20	6
5	300	30	20	6
6	800	15	40	6
7	300	60	40	6
8	1500	60	40	6
9	300	15	0	10
10	1500	30	0	10
11	800	60	20	10
12	1500	15	40	10
13	300	15	0	20
14	1500	15	0	20
15	800	30	0	20
16	300	60	0	20
17	1500	60	0	20
18	800	15	20	20
19	1500	30	20	20
20	300	15	40	20
21	1500	15	40	20
22	300	60	40	20
23	1500	60	40	20
24	1500	60	40	20
25	1500	60	40	20
26	1500	60	40	20

**Table 2:** List of independent variables with their feasible investigational ranges that were used in optimisation DOEs.

Independent variable	Level		
	High level	Medium level	Low level
Processing vessel speed (rpm)	1500	800	300
Processing time (min)	60	30	15
Air pressure (psi)	40	20	0
Batch Size (g)	20	10	6

#### DOE model analysis

After model fitting, model verification was carried out to ensure its validity and reproducibility through sequential elimination of the insignificant runs using distance to model plots while evaluating the effect of the elimination on the model using lack of fit plots. Furthermore, the model terms were reviewed individually and those with no statistical significance were eliminated because their influence on the values of CQAs was negligibly small [10]. Two eliminations were carried out; the insignificant runs and the insignificant model terms.

ANOVA analyses for total variations of the responses at two levels were carried out (variances attributed to regression model and variances obtained from residuals and replicate errors). The *p* value for the significance of the regression coefficient was set at less than 0.05, while the model error or lack of fit was set at a value of bigger than 0.05 indicating the insignificance of the model error.

A statistical model comprising both interaction and polynomial terms was used to evaluate the responses and it was displayed according to Eq 2 for each response [10,23]:

$$y=\beta_{0}+\beta_{1}x_{1}+\beta_{2}x_{2}+\beta_{3}x_{3}+\beta_{11}x_{1}^{2}+\beta_{22}x_{2}^{2}+\beta_{12}x_{1}x_{2}+\beta_{13}x_{1}x_{3}+\beta_{23}x_{2}x_{3}\ldots +\varepsilon$$ Eq (2)

Where (*y*) is the modelled response, *B*_0_ is the arithmetic mean response of the runs; *β*_*1*_, *β*_*2*_, *β*_*3*_, *β*_*11*_, *β*_*22*_, *β*_*12*_, *β*_*13*_, *β*_*23*_ are the estimated coefficients for the main effects (*x*_*1*_, *x*_*2*_, *x*_*3*_); the polynomial terms (*x*_1_^2^, *x*_*2*_^*2*^) and the interaction terms (*x*_*1*_*x*_*2*_, *x*_*1*_*x*_*3*_ and *x*_*2*_*x*_*3*_) respectively. **ε** is a constant [24,25]. The final regression model equations were determined upon the evaluation of individual terms and elimination of all insignificant terms. Once the model was significant, evaluation and prediction tools were used to identify the effect of individual or interaction CPPs on CQAs and the results used to predict design space.

## Results and discussion

Initial screening studies targeted the identification of CPPs and CQAs that will be further optimised using QbD. Therefore, following the initial screening experiments, the tree diagram (Fig 1) served as a template for the development of an optimisation model for the dry particle coating process. The study discusses the risk assessment (criticality) carried out for CPPs and CQAs followed by DOE model verification, ANOVA and main/interaction effects on CQAs. Finally, the prediction part where the design space meets the requirements of CQAs was set.

### Screening studies

**Fig 3:**
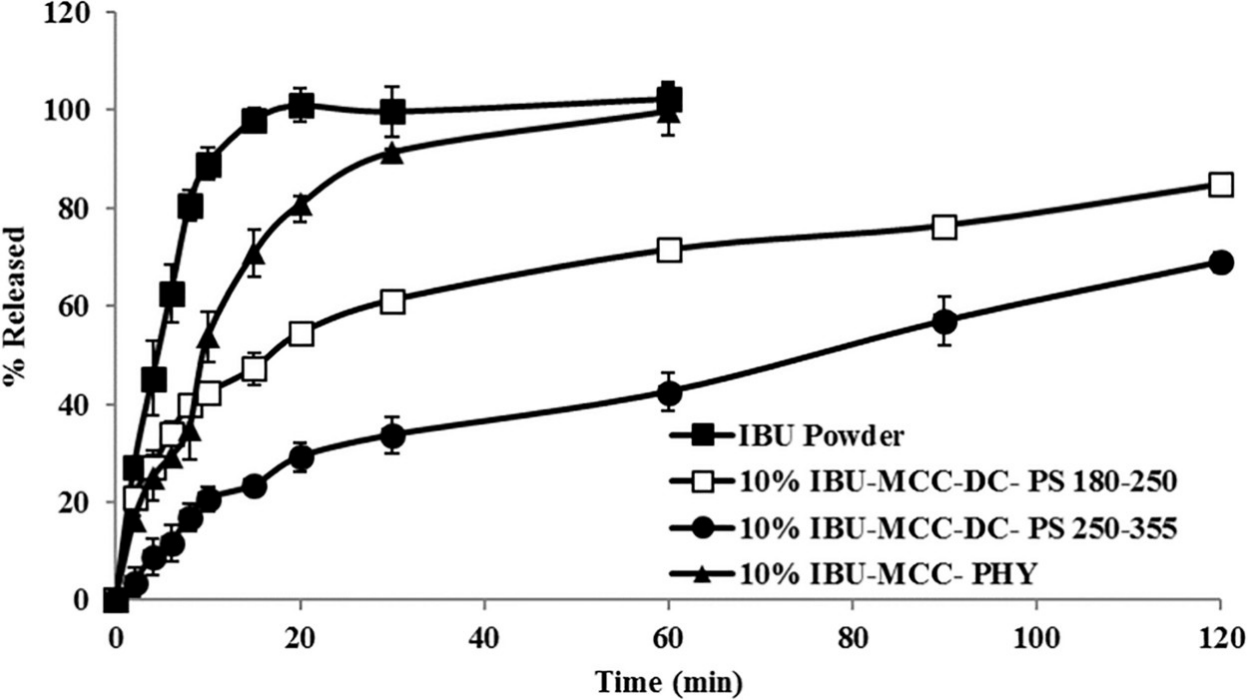
Comparison of dissolution profile of 10% w/w (▲) ibuprofen (IBU) with MCC physical mixture (PHY), dry coated particles (DC) with different carrier particle size (PS) (□) 180–250 μm and (●) 250–355 μm, and (■) ibuprofen (IBU) powder determined using USP Basket Method at 50 rpm measured in phosphate buffer pH 7.2 at 37°C, (mean ±SD, n = 3).

Ibuprofen was selected as a model active pharmaceutical ingredient (API) owing to its low solubility (21 mg/L) and availability as a cohesive powder with small mean particle size [26]. MCC was chosen as model carrier particle as it is a widely-used excipient and is available in a range of particle sizes. Dry particle coating process requires an initial de-agglomeration of the cohesive powder’s guest particles into their primary particles via the application of external forces (convection, diffusion and shear forces), producing a homogenous blend. To obtain FP where the fine guest particles were attracted to the surface of the carrier, additional shear forces need to be introduced into the blending system using the dry coating device. MCC particles were dry coated with 10%w/w ibuprofen, followed by assessment of the blend for dissolution. Fig 3 shows the dissolution profile of the dry coated powder, physical mixture and ibuprofen powder. The results revealed a significant difference in the release profile (one way ANOVA, p<0.0001) between the dry coated powder and the physical mixture particularly after 15 minutes. The physical mixture showed dissolution rate that was slower than that of the powder alone (more than 90% within 30 minutes), whereas the dry coated blend took 2 hours to reach 85% release. The initial fast dissolution of particles from the dry coated blend was possibly due to the existence of dispersed ibuprofen particles within the system that were not attached to the carrier surface (i.e., non-adsorbed particles) which result in immediate dissolution due to the large surface area whereas the dry coated particles account for the sustained release pattern. The slow release from the dry coated blend compared to physical mixture, could be attributed to the strong adhesion of the ibuprofen particles to the insoluble carrier, whereas the release of ibuprofen from the physical mixture was similar to those of interactive mixture which showed higher dissolution rate than that of dry coated mixture [27–30].

Increasing the carrier particle size (250–355 μm) resulted in further reduction in the release rate of ibuprofen. The efficiency of powder coating increased with the increase in the ratio between the carrier and guest particle size [17]. The dissolution data showed a statistically significant reduction in dissolution rate over the period of 2 hours compared to that of smaller carrier particle size (180–250 μm) (one-way ANOVA, p<0.0001). These results were consistent with previously reported results that the particle size of the guest and carrier plays a vital role in dry coating process. According to those studies, the attraction force between guest and carrier increases with the increase in the ratio between the size of guest to carrier particles [30–32].

#### PSA results

The results of PSA as depicted in Table 3 showed that the VMD for ibuprofen was 25.4 ± 1.24 μm and median (X_50_) was 22.88 ± 1.07 μm. 90% of the ibuprofen particles were below 47.78 ± 1.89 μm and hence the largest proportion of the powder mix was made of fine powder.

**Table 3:** Particle size distribution parameters for ibuprofen (IBU), MCC and the various mixtures (Dry coated (DC) and Physical (PHY)) using laser diffraction technique, (mean ±SD, n = 3).

Ingredient / or % w/w IBU in MCC	X_10_ (μm)	X_50_ (μm)	X_90_ (μm)	VMD (μm)
**MCC (180–250 μm)**	41.75 ± 3.88	119.69 ± 4.52	160.28 ± 1.76	112.1 ± 4.38
**IBU (38–53 μm)**	6.54 ± 0.66	22.88 ± 1.07	47.78 ± 1.89	25.4 ± 1.24
**5% IBU (DC)**	68.53 ± 33.40	130.14 ± 1.03	164.38 ± 0.32	122.11 ± 2.98
**5% IBU (PHY)**	51.39 ± 32.05	130.05 ± 1.28	164.41 ± 0.39	120.97 ± 3.26
**10% IBU (DC)**	46.79 ± 5.68	130.97 ± 3.1	164.72 ± 2.12	122.71 ± 3.29
**10% IBU (PHY)**	29.38 ± 10.11	126.32 ± 1.29	163.09 ± 1.29	115.11 ± 4.47

As for MCC with particle size ranging between 180–250 μm obtained from sieving, laser diffraction particle size analyser results produced an incomplete distribution curve as the powder contained particles with size exceeding the 200 μm limit (limitations of the equipment). However, this process was used to assess the fines within the powder and for MCC (180–250 μm) less than 10% of the powder had particle size smaller than 41.75 ± 3.88 μm. The laser diffraction method produces high air jet aided by compressed air that results in dispersion of the sample and therefore, it is expected that the agglomerates originating from fine powder were dispersed into primary particles and the percentage of fine is a true representation of non-dry coated drug particles. Similar results were reported [33].

On further characterisation of the powder mixtures for both dry coated and physical mix, the results of analyses demonstrated that dry coated mixtures with 5% w/w and 10% w/w ibuprofen load had less fines compared to physical mix indicating the adherence of ibuprofen fine powder onto the surface of MCC. The use of this test as a quick qualitative indication of the formation of coated particles was further investigated in the optimisation study.

#### FTIR results

**Fig 4:**
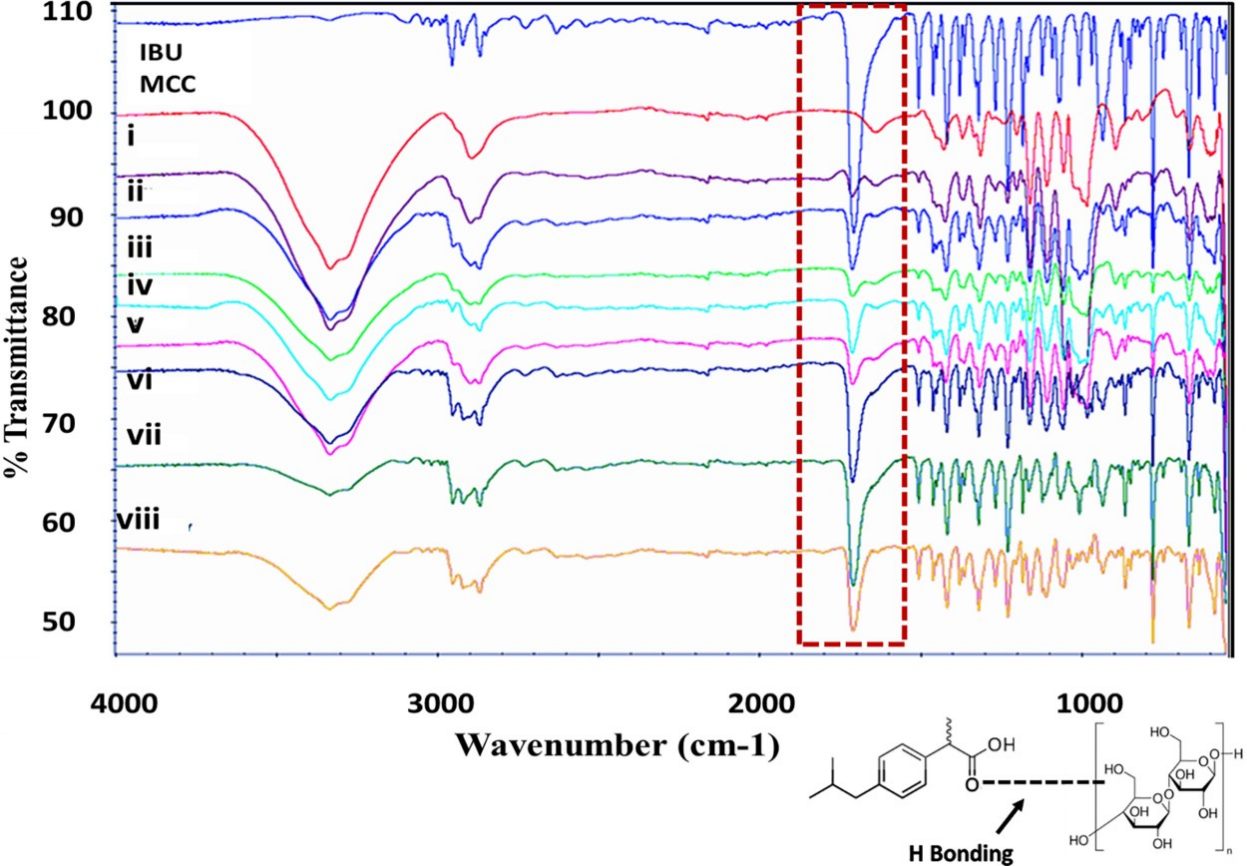
FTIR spectra from top to bottom of ibuprofen (IBU) powder, MCC powder, (i) 2%w/w IBU in MCC coated mixture, (ii) 2%w/w IBU in MCC before coating, (iii) 5%w/w IBU in MCC coated mixture, (iv) 5%w/w IBU in MCC before coating, (v)10%w/w IBU in MCC coated mixture, (vi) 10%w/w IBU in MCC before coating and (vii) 20%w/w IBU in MCC coated mixture, (viii) 20%w/w IBU in MCC before coating highlighting the reduction in the intensity of the peak at wave number 1708 cm^-1^ (labelled with red rectangle) of ibuprofen that is attributed to C = O due to the formation of hydrogen bonding between the hydroxyl (OH) and (C = O) groups. Lower diagram highlighting the hydrogen bonding formation between ibuprofen and MCC.

FTIR of ibuprofen powder showed absorption peaks that tally with the frequencies of vibrations between the bonds within the molecule. The peak size in each spectrum is a direct indication of the amount of the material available within the sample. Further, hydrogen bonding can be identified by studying the differences in the extent of absorbance [21]. The FTIR spectra of ibuprofen (Fig 4) shows band at 2954.24 cm^−1^ which can be assigned to CH_3_ asymmetric stretching. Similarly, peaks with very high intensities were recorded at 1708.27 cm^−1^ and 1230 cm^−1^ owing to C = O stretching and C-C stretching respectively. Another band was observed at 779 cm^−1^ which was attributed to CH_2_ vibration. The above bands represent the main bands providing the finger print for ibuprofen [34,35]. The FTIR spectra for MCC (Fig 4) showed two strong bands at 3301 cm^-1^ and at 1644 cm^-1^ corresponding to stretching and bending of the surface hydroxyl groups. An additional strong peak was shown at 2895 cm^-1^ due to the asymmetric stretching vibration of C-H of the pyranoid ring [36,37].

The FTIR spectra of the dry coated particles and physical mixtures at various concentrations of ibuprofen ranging from 2%w/w to 20%w/w are depicted in Fig 4. From the spectra, it can be noted that there was no change to the main peaks associated with ibuprofen or MCC with both dry coated and physical mixtures at all concentrations. The trough at wave number 1708 cm^-1^ of ibuprofen that is attributed to C = O stretching was present in all mixtures and did not change its position. On the other hand, a reduction in the intensity in carbonyl band of ibuprofen dry coated mixtures compared to physical mixtures was observed. It is possible that the change in intensity was due to the formation of hydrogen bonding between the carbonyl group of ibuprofen and the hydroxyl group of MCC (see Fig 4). Similar findings were reported by [34] who investigated the use of FTIR to study the formation of hydrogen bonding between carbonyl group of acrylic esters and OH groups of hexanols and cresols, where the intensity of spectra representing carbonyl group (C = O) was reduced due to the formation of hydrogen bonding between the hydroxyl (OH) and (C = O) groups.

Ibuprofen is available as a cohesive powder where every crystal unit is made of four ibuprofen molecules that are attached with hydrogen bonding (two) leaving two remaining carboxylic–groups ready for further hydrogen bonding with adjacent neighbouring units [38]. As MCC has multiple OH groups, it is possible that hydrogen bonding has formed between the H of the OH group of MCC and the carbonyl O of ibuprofen (see diagram in Fig 4). FTIR spectra revealed no shift in any of the peaks that correspond to ibuprofen or MCC. There was merging of the troughs related to IBU (2954- CH3 group) and the MCC (3301- OH group) as they appear at almost the same wave number range. However, since the intensity of the peak is related to quantity of the material, additional reference peak was considered (the trough at 1230 cm^−1^ owing to C-C stretching). The intensity of this trough (at 1230 cm^-1^) was divided by the intensity of the trough at 1708.27 cm^−1^ and the ratio was obtained. Results showed that there was a reduction in the ratio when dry coated particles were compared to the physical mixture, thus confirming that the change was not related to the quantity of ibuprofen.

Pfeffer and colleagues presented changes in FTIR spectra as a mean to identify mechano-chemical surface reaction between cellulose or corn starch and silica (used as guest). The absorbance in the -OH region was reduced due to the interaction of cellulose with the OH group in silica with loss of a water molecule [31].

### Process optimisation

Based on the screening results, the main process variables to be optimised were processing speed, gas pressure, processing time and batch size. The CQAs obtained from the screening experiments were content uniformity, API release at 60 minutes, X_10_ following PSA and the intensity of the FTIR spectrum at 1708 cm^-1^ of ibuprofen. Process optimisation included multiple steps namely; setting the specifications of the CQAs, followed by risk assessment and DOE study.

#### CQAs

CQAs of dry coated particles were identified based on literature, compendia requirements and initial screening experiments [6,8]. Table 4 lists the CQAs were identified from the QTPP based on the impact of each parameter on the production of modified release FP.

**Table 4:** CQAs of the final powder dry coated blend.

CQA	Targeted outcome	Comments
Content Uniformity	Conforms with the BP and USP <905> specification (95–105%), RSD ≤5% [20,39].	Compendia requirement and deviations from the targeted content is a direct indication of product failure [20,39].
Dissolution	Percentage release of API is < %85 after 60 minutes.	Failure to reduce the dissolution rate of API is a failure to produce modified release FP and therefore an indication of process failure (physical mixture is obtained rather than dry coated FP).
Particle size	Increase in the size of particles in the X_10_ region of the cumulative particle size analysis.	The reduction in particle size in X_10_ region indicates the presence of fines and that the anticipated FPs were not formed.
Intensity of FTIR spectrum at 1708 cm^-1^ of ibuprofen that correspond to C = O stretching.	Reduction in the spectrum intensity.	An indirect indication of the formation of hydrogen bonding between ibuprofen fine guest particles and coarse MCC carrier particles between C = O group in ibuprofen and OH groups in MCC [34].

#### Risk assessment for CQAs and process variables

Risk assessment looked at the effect of every input and process parameter on individual CQA which would enable the determination of independent factors (process variables)) that were used in the DOE experimental design (Table 5). Risk ranking was based on either compendia requirement, initial screening results or literature. Process parameters with risk ranking ranging between medium and high were taken as the factors in the DOE.

**Table 5:** Risk assessment of the input and process parameters against the CQAs with relevant justification to produce FP with extended release profile.

Process Variables	CQAs				Justification
	Content Uniformity	Dissolution	PSA (X_10_)	Bond formation	
**Speed**	High	High	High	High	High speed, air pressure and processing time have direct impact on the extent of powder blending that would lead to an increase in force that aids mixing and formation of FP [40]. As the carrier is insoluble, dissolution rate would be hindered upon formation of FP, which implies reduction in fines through the increase in X_10_ value and reduction of FTIR spectra due to formation of hydrogen bonding [34,40].
**Air Pressure**	Medium	High	High	High	
**Processing time**	High	High	Medium	Medium	
**Batch size**	Medium	High	High	High	The increase in batch size beyond the mixing capacity of the processing vessel might not allow enough space for complete de-agglomeration collision and dry particle coating [40].

#### Design model creation

Once the process variables and responses (CQAs) were established, the software developed the candidate model as the D-optimal with 26 experiments that were carried out according to the randomisation order set by the software, of which 4 experiments represented replicates necessary to ensure model reproducibility. The compiled worksheet was then fitted into the D-optimal model. Once the model was fitted, model verification and regressions equations to enable understanding of the effect of process variables on CQAs were conducted. All insignificant model terms were removed from the model equation.

#### Model verification

**Fig 5:**
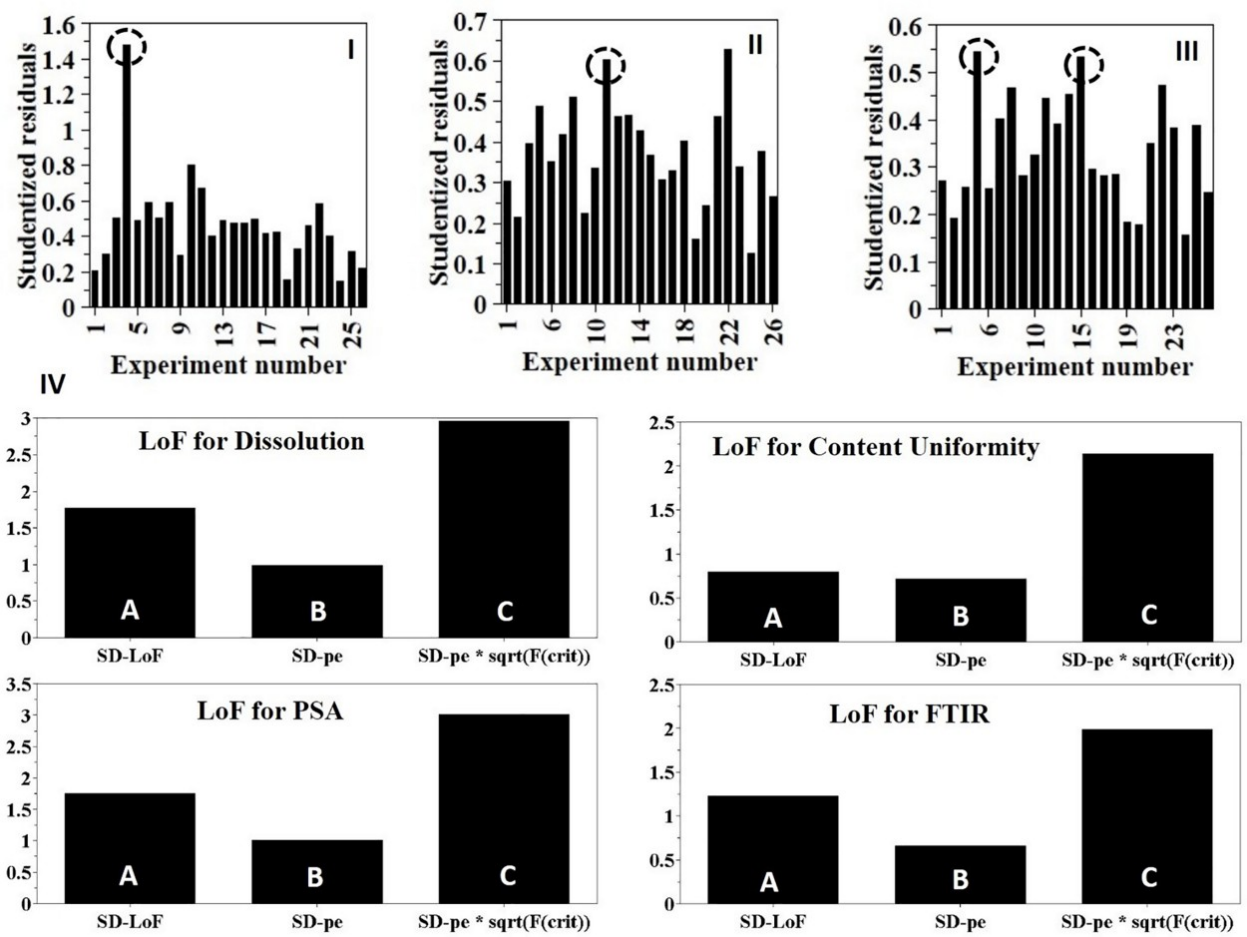
(I,II &III) Distance to model (Y) plot of individual runs to identify insignificant runs (highlighted with circles). Note that the distance to model changes upon removal of the furthest run. (IV) Lack of Fit plot showing standard deviation (SD) due to lack of fit (A), SD of pure error (B) and SD of pure error* critical F-value (C) for the four responses. If A ≤ C, the model shows no lack of fit.

Model verification was carried out using distance to model plot (Fig 5I–5III). A total of 22 included and 4 excluded runs: initially, run 4 (Fig 5I) was the farthest from the model followed by run 11 (Fig 5II) then finally the last two run 5 and run 15 (Fig 5III) were removed.

Once the insignificant runs were excluded, the model fitness was investigated using the lack of fit plot (Fig 5IV) that is denoted by three bars for each CQA. The first bar (A) represents the standard deviation of the lack of fit that is related to the model error. The second bar (B) represents the pure error. The third bar (C) is the standard deviation of the pure error multiplied by the critical F-value at the 95% confidence interval. To assess lack of fit, the standard deviation due to lack of fit (A) was compared with the standard deviation bar* F-value (C). As shown in Fig 4, for all responses, the value of standard deviation due to lack of fit (A) was below the standard deviation*F-value (C) which confirms model validity.

The quadratic model was then fitted using the 12 terms for each response including; constant, 4 single terms (speed, time, air pressure and batch size), one quadratic term (speed^2^) and six interaction terms (speed*time, speed*air pressure, speed*batch size, time*air pressure, time*batch size and air pressure*batch size).

**Fig 6:**
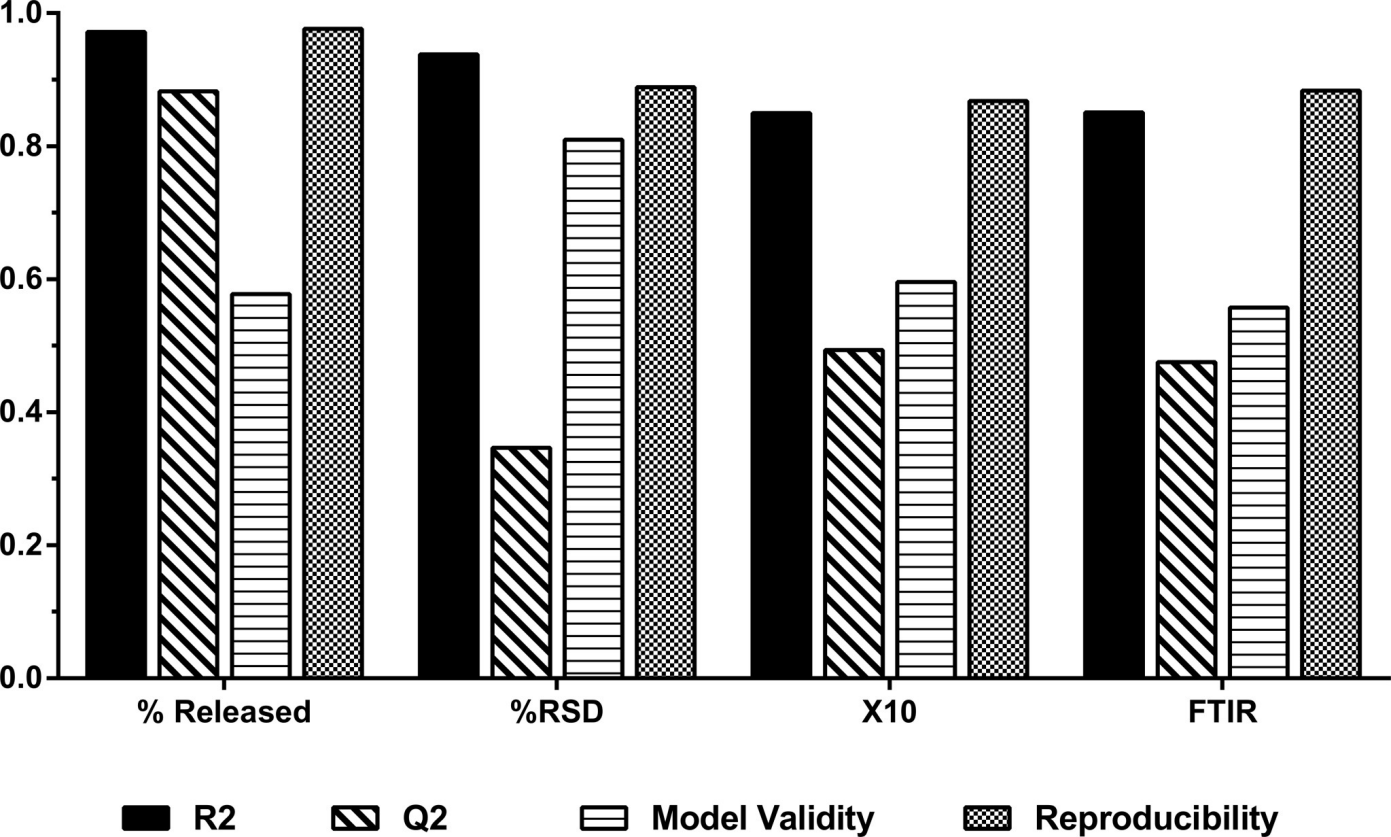
Summary plot of the four responses (CQAs) of fit showing model Fit (R2), predictability (Q2), model validity and reproducibility. Model fitted with PLS.

The results of every response are depicted in Fig 6. R2 and Q2 values gives the best estimate to the fitting of the model with R2 is the percentage of variation of the response and the degree of how the data fits the model. Q2 represents the prediction ability of the model (how well the model will predict new data). Overall the model fitted well all the four responses with high R2 value of 0.973 for dissolution, 0.944 for content uniformity, 0.851 of PSA and 0.859 for FTIR. The predictive power varied among responses; for dissolution, almost 88.2% of the 97.3% fitting, could be predicted. In an optimisation process, high Q2 of responses is expected as the ultimate purpose is to optimise and predict the model behaviour. The model showed that the predictive power of content uniformity was at the acceptable range (> 0.25). Out of 94.4% of fitted results, 34.6% could be predicted while any noise responsible for reducing Q2 could be attributed to the sampling process. As for particle size, the model fitted with 85.1% accuracy (very good model) of which, 49.4% could be predicted. The more FP produced, the lower the amount of fine and hence the larger the particle size at the X_10_ region. FTIR spectrum transmittance intensity for ibuprofen at C = O link represents good fitting in the model with 47.5% out of 85.9% of the results could be predicted. As for validity, all responses were valid as they exceeded the threshold of 0.25 (16). Validity values ranged from the lowest for 0.577 (FTIR), 0.591 (dissolution), 0.598 (PSA) to the highest value for content uniformity (0.837). Reproducibility was very high for all responses (> 0.5 threshold) indicating good control over experiment and low pure error. The reproducibility ranged from 0.976 for dissolution, 0.889 for content uniformity, 0.868 for PSD, to 0.883 for FTIR.

#### Analysis of variance (ANOVA) results

Table 6 summarises the results obtained from ANOVA analysis, where in the current investigation, the p value was <0.05 for all CQAs suggesting that the regression model is statistically significant for all responses. Secondly, the model error due to lack of fit was insignificant as the p value was more than 0.05 for all the responses.

**Table 6:** Summary of results obtained from ANOVA of the four responses to test model validity. *P* is probability and R2 is the regression coefficient.

	*P*	R2
**Dissolution**		
Regression	<0.00001	0.97
Lack of Fit	0.195	
**Content Uniformity**		
Regression	<0.00001	0.944
Lack of Fit	0.522	
**PSA**		
Regression	0.007	0.851
Lack of Fit	0.201	
**FTIR**		
Regression	0.006	0.859
Lack of Fit	0.184	

#### Regression model equations for CQAs

**Fig 7:**
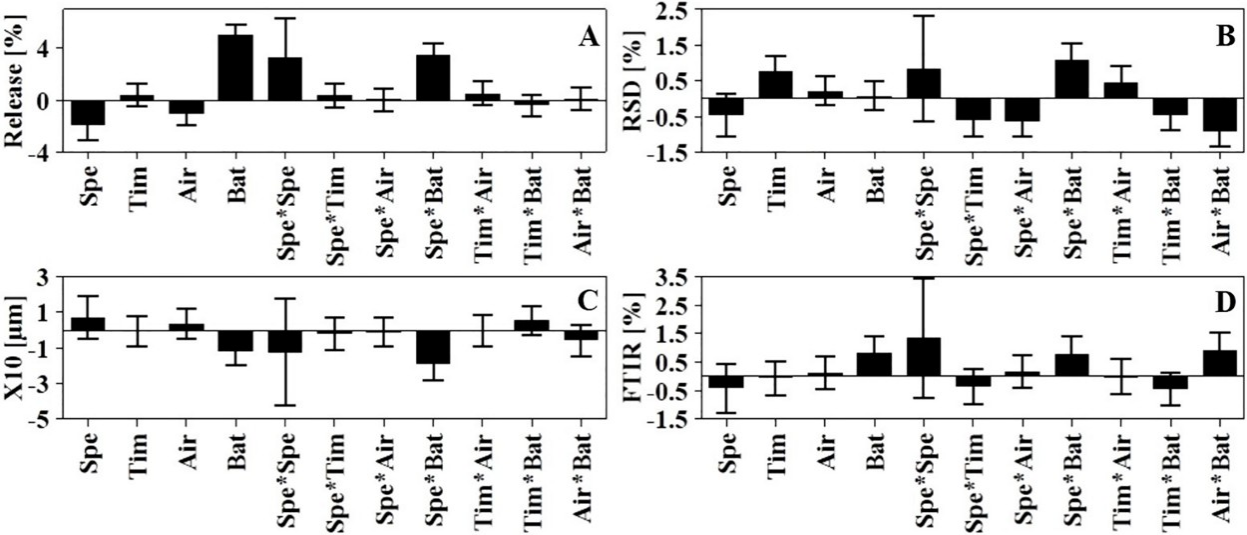
Regression coefficient plot of dry particle coating process model terms for each CQA (response). (A) % release in 60 minutes, (B) %RSD, (C) X_10_ value (μm) and (D) FTIR spectrum.

Once the significance of the CQAs within the model was established; the regression coefficients for all the model terms were investigated to identify the significant model terms per CQA using regression coefficients plot (Fig 7). The size of the coefficient (the length of the bar) indicates the effect of the factor on the response whereas direction of the coefficient represents the negative or positive impact on the response. The coefficient will be considered significant when the confidence intervals do not cross zero. The first four coefficients (the linear terms) disclose the main effect of the each CPPs. The ones that follow, also called interaction terms, reveal interactions (if any) within the factors. The size of the coefficient is the real effect while the confidence interval represents the noise [10]. For example, the most significant factors affecting dissolution were batch size followed by speed then air pressure whereas speed*batch size was the most significant interaction term. The quadratic term speed*speed had high significance.

The proposed regression model for every CQA was then built based on the significant correlation coefficients for each independent factor (Fig 6) and the effects they had. For each response, the quadratic model entailing 12 model terms was fitted, only those with significance were included in the regression equations. The fitted equations for all responses are displayed in (Eq 3–6).

$$Y_{1}=92.15-4.23\;X_{1}-1.92\;X_{3}+10.83\;X_{4}+6.92\;X_{1}^{2}+7.78\;X_{1}X_{4}$$ Eq (3)

$$Y_{2}=1.85+1.54\;X_{2}-1.08\;X_{1}X_{2}-1.4\;X_{1}X_{3}+2.47\;X_{1}X_{4}+0.93\;X_{2}X_{3}-1.18\;X_{2}X_{4}-2.06\;X_{3}X_{4}.$$ Eq (4)

$$Y_{3}=19.86-2.58\;X_{4}-4.31\;X_{1}X_{4}$$ Eq (5)

$$Y_{4}=13.59+1.81\;X_{4}+1.82\;X_{1}X_{4}+2.0\;X_{3}X_{4}$$ Eq (6)

From the equations, Y_1_ is the dissolution results (percentage released after 60 minutes), Y_2_ is the content uniformity measured as relative standard deviation (RSD), Y_3_ is the results from PSA expressed as the particle size at the X_10_ region and Y_4_ is the FTIR results presented as intensity of the spectra at 1708 cm^-1^. X_1_ is the speed (rpm), X_2_ is processing time (min), X_3_ is air pressure (psi) and X_4_ is the batch size (g). The value of each coefficient indicates the impact; the higher the value the more the effect. The sign of the coefficient provides information on the effect being positive or negative. Positive sign with batch size for example of (10.83) has a positive effect on dissolution rate. Whereas speed (-4.23) demonstrated the most significant negative impact factor where increasing the speed resulted in a reduction of dissolution rate.

#### Effect of independent factors on CQAs

There is a close association between dissolution, PSA and FTIR results. Theoretically successful FP should result in reduction of dissolution rate, increase in the particle size at the X_10_ region of the powder mix and finally and reduction in the intensity of FTIR spectra at 1708 cm^-1^ due to the formation of hydrogen bonding. As such, the effect plots for this group will be discussed concurrently.

#### Effect on dissolution, PSA and FTIR

**Fig 8:**
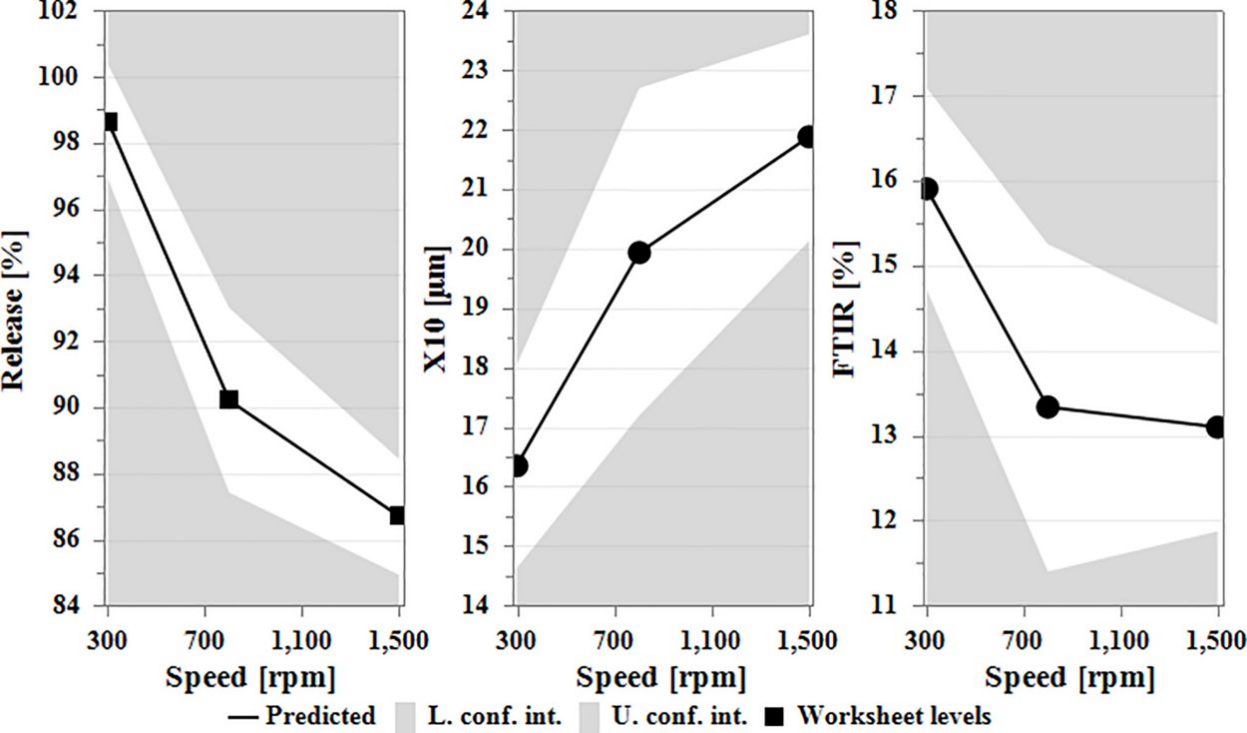
Main effect plot of batch size on the dissolution, PSA and FTIR spectrum when other factors maintained at their middle values. Note the D-optimal design has no centre points and hence were not displayed.

The most significant single model factor affecting production of FP was batch size (Fig 8). The collective effect on the three responses (dissolution, PSA and FTIR) showed a negative impact on the formation of particles. The increase in batch size resulted in an increase in dissolution, reduction of X_10_ fraction of particle size analysis and an increase in the FTIR spectrum intensity (1708 cm^-1^).

**Fig 9:**
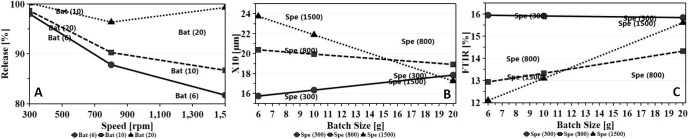
Interaction effect plot of speed with increasing batch size on (A) dissolution rate (B) PSA and (C) FTIR spectrum while the other factors were maintained at their middle values.

The analysis (Eqs 3, 5 and 6) also showed that speed and batch size interaction term had an antagonistic effect on dissolution (Fig 9A). When increasing the speed, the dissolution profile was reduced. However, the influence of speed was greater when the batch size was set to its lowest level. The increase in speed resulted in higher shear force and hence production of FP with reduced dissolution rate. However, when the batch size exceeded 10 g, an antagonistic effect on dissolution with a dissolution rate reaching 100% within 60 minutes resembling the physical mix obtained at 300 rpm was noted. The interaction predictive model showed that the effect of speed was further enhanced when the rotational speed exceeded 800 rpm.

The interaction between speed and batch size was the most significant on PSA as evident from the regression model (Eq 5) with coefficient factor of -4.31. From Fig 9B, a small batch size with high speed resulted in larger particles. The fine guest particles got strongly attracted to the coarse carrier particles as evidenced by an increase in the X_10_ particle size. This was attributed to the formation of not only van der Waals forces (responsible for adhesion of fine particles to large carrier particles) [33,40] but also hydrogen bonding between hydrogen of the MCC and the C = O of ibuprofen which was evident from the reduction in the intensity of the FTIR spectra at 1708 cm^-1^. However, with the increase in batch size, the effect of speed declined resulting in an increase in the fines.

**Fig 10:**
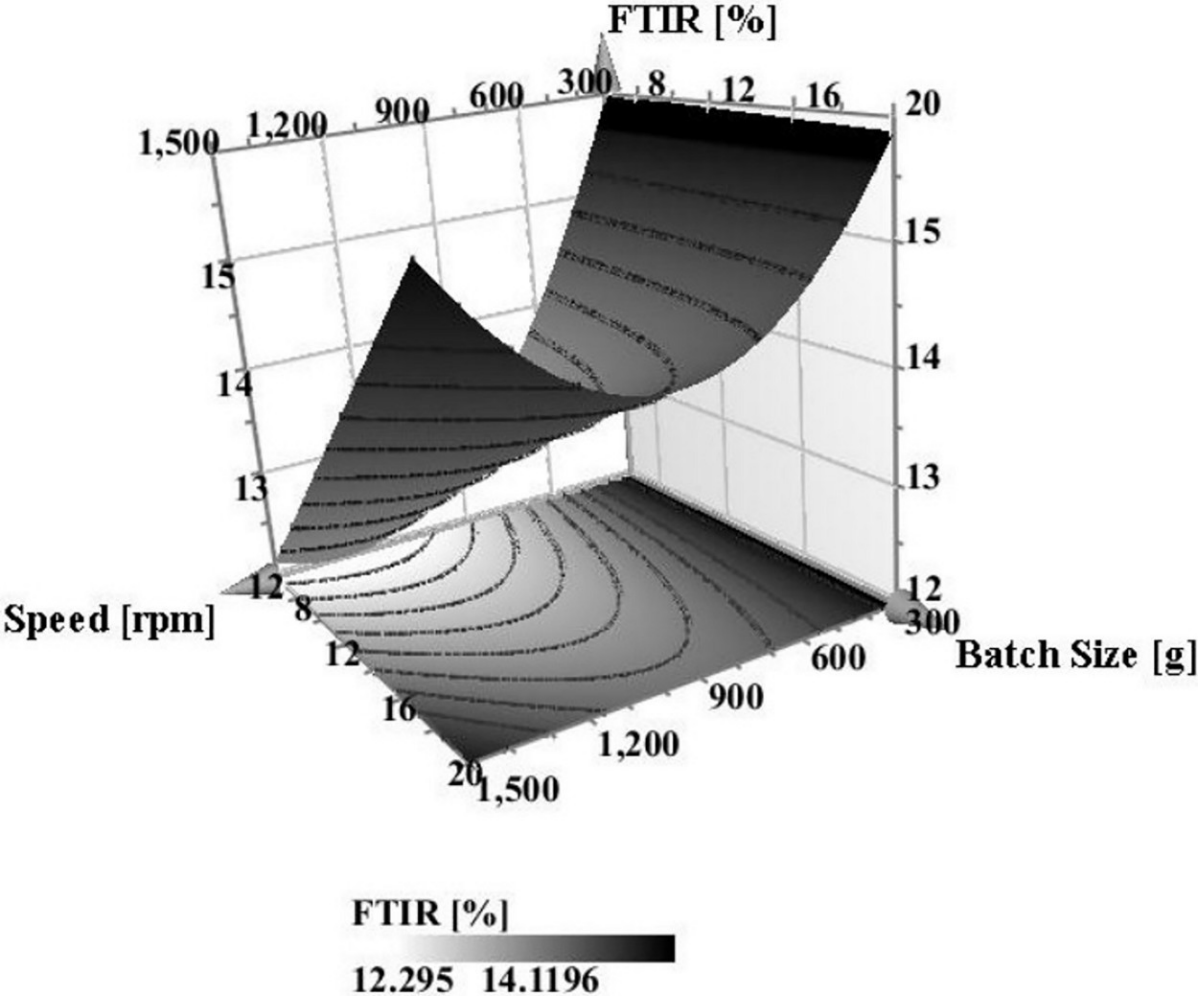
Response Surface Model (RSM) Plot of the effect of speed and batch size on the FTIR of the powder mixture while maintaining air flow and time at their middle values (mid-region highlighted the interaction effect of both factors).

Likewise, speed and batch size as interaction model terms had an effect on FTIR (Fig 9C) that even clearly demonstrated from Fig 10 showing the interaction region on the RSM plot. At low speed, FTIR spectrum intensity was at its maximum due to absence of any hydrogen bonding. At low speed, the batch size had no influence on FTIR and the device was possibly working as a powder blender. However, with the increase in speed and reduction in batch size, there was a significant reduction in the intensity of the FTIR spectrum suggesting the formation of hydrogen bonds with the resultant particles.

Interestingly, the effect of the quadratic term (speed^2^) on dissolution rate showed a positive value (Eq 3). The positive sign indicates a convex curve [41]. It was therefore assumed from the model that initially, with the increase of speed, the force produced in the system resulted in the formation of FP which negatively impacted dissolution. However, beyond a limit, the increase in speed may result in rapid collision of particles above the limit of attract ion which could result in detachment of the guest particles from the surface of the host particles resulting in attrition and could possibly enhance the dissolution rate.

#### Effect on content uniformity

**Fig 11:**
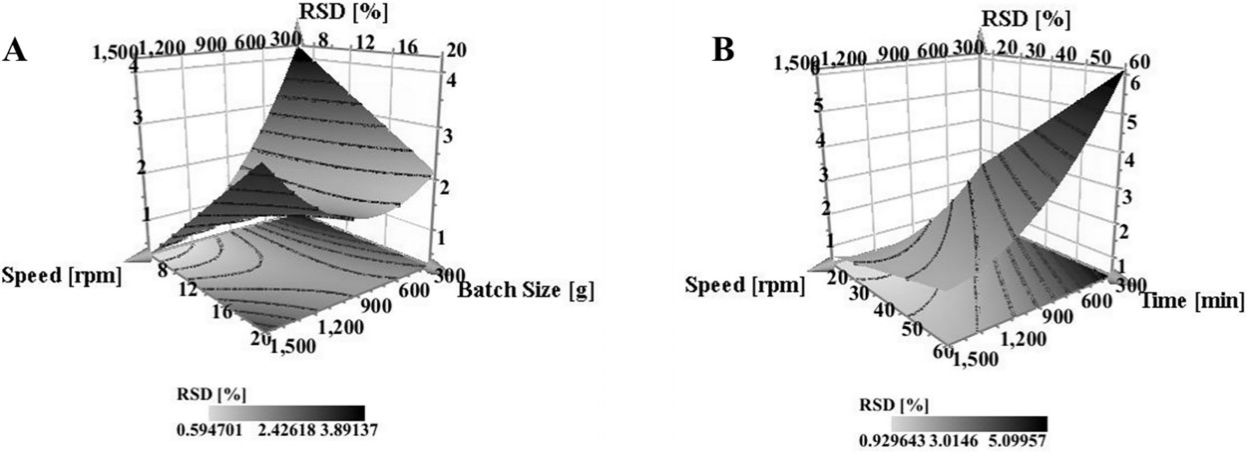
Response Surface Model (RSM) Plot of the **(A)** effect of speed and Batch size on the content uniformity of ibuprofen in the powder mixture while maintaining processing time and air flow at their middle values, **(B)** the effect of speed and processing time on the content uniformity of ibuprofen in the powder mixture while maintaining air flow and batch size at their middle values.

Low RSD in content uniformity studies is an indication of blend homogeneity once the content is within the established range of 95–105%. Based on the regression equation (Eq 4) RSD was affected by many interaction terms however, speed and batch size had the highest negative impact (regression coefficient +2.47) with a positive sign indicating an increase in RSD. At low to moderate speed the increase in batch size has limited effect on content uniformity of the mixture. Contrary to that, at high speed the smaller the batch size the lower the RSD and hence the better the homogeneity. This could be attributed to the effect of shear force resulting in de-agglomeration and proper dispersion of the fine powder within the mixture. The RSM plot (Fig 11A) shows the extent of interaction between speed and batch size and enables the prediction of the best working space, where homogenous mixture with RSD<2% was obtained with speeds exceeding 800 rpm while maintaining the batch size below 10 g. As the target of this experiment was to identify the optimal process parameters to produce FP while maintaining homogeneity, the optimal working parameters selected for FP production will result in a homogenous mixture with RSD far below the proposed 5%. The interaction between speed and processing time (Fig 11B) showed that the RSD was reduced with the increase in speed and reduction in time. Zheng and colleagues [42] reported that mixing for extended duration may undesirably affect the content uniformity of the mixture. However, as the effect of processing time was decreased with the increase in speed, above 500 rpm the RSD was far below 1%. At a speed of 1500 rpm set for the maximum processing time of 60 minutes, the resultant RSD was 1.4% whereas reduction of processing time to 15 minutes produced RSD less than 0.5%.

**Fig 12:**
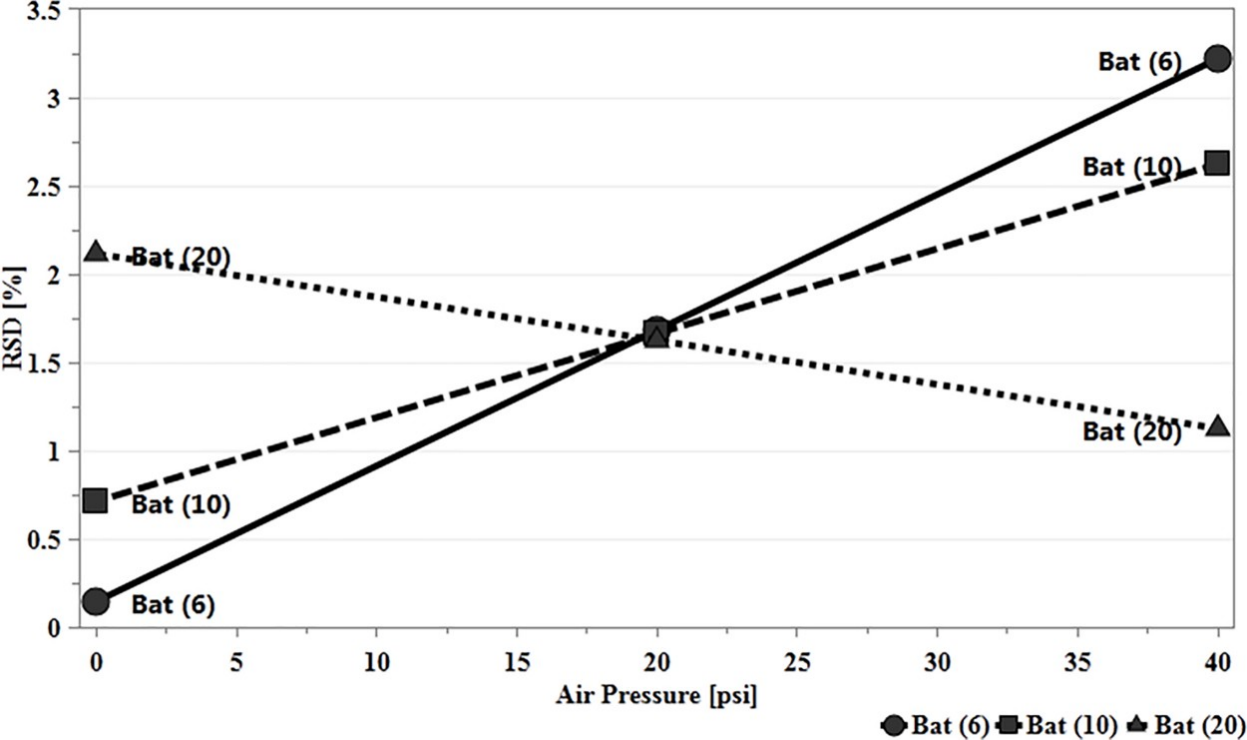
Interaction effect plot of batch size with increasing air flow on content uniformity while other factors were maintained at their middles values.

From Eq 4, the second most significant interaction term affecting content uniformity was batch size and air pressure. Low RSD was best obtained when working at the low limits of both batch size and air pressure, nevertheless, increasing air flow produced a lower RSD with batch size set at its maximum limit. In all cases the change in RSD did not exceed 2%. Since FP were best obtained at lower batch size with moderate to high air pressure, such working conditions could be identified through Fig 12.

**Fig 13:**
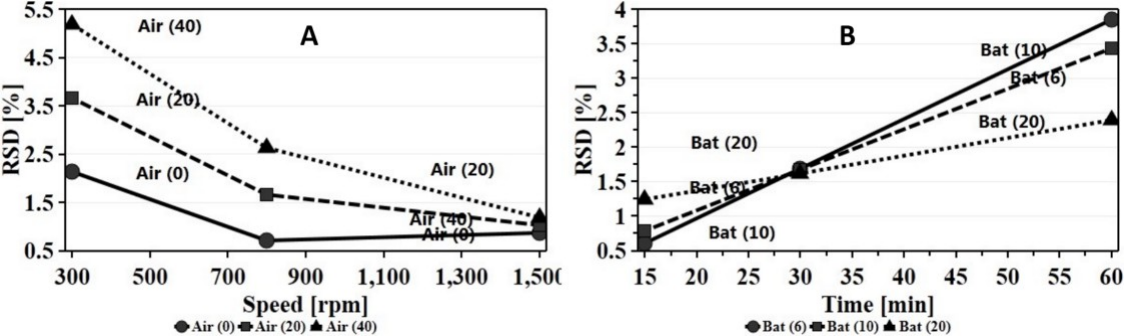
Interaction effect plot of (A) air flow with increasing speed (B) batch size with increasing processing time on content uniformity while other factors were maintained at their middle values.

Speed of mixing enhanced homogeneity when mixing occurred at lower air pressure. The antagonistic effect of air flow was observed when the speed was at its low limit (Fig 13A). This could be attributed to induction of turbulence in the system by air flow that might cause fine particles to adhere to the surface of the container particularly if the force produced (diffusion and convection currents) was not enough to de-agglomerate the cohesive ibuprofen particles and adhere it over the carrier particles [42].

Processing time with increasing batch size was an interaction type of relationship; at low processing time the mixture was more homogenous with a lower RSD when the batch size was set within the small range. Interestingly this occurred when the speed and air flow were set at their average. This could be attributed to the formation of FP. It was expected that the blend of FP aids homogeneity and as discussed earlier the smaller the batch size the higher the possibility of producing a hybrid mixture. However, it is worth mentioning for these two factors that the highest RSD obtained did not exceed 4% (Fig 13B).

#### Setting the optimal zones

**Fig 14:**
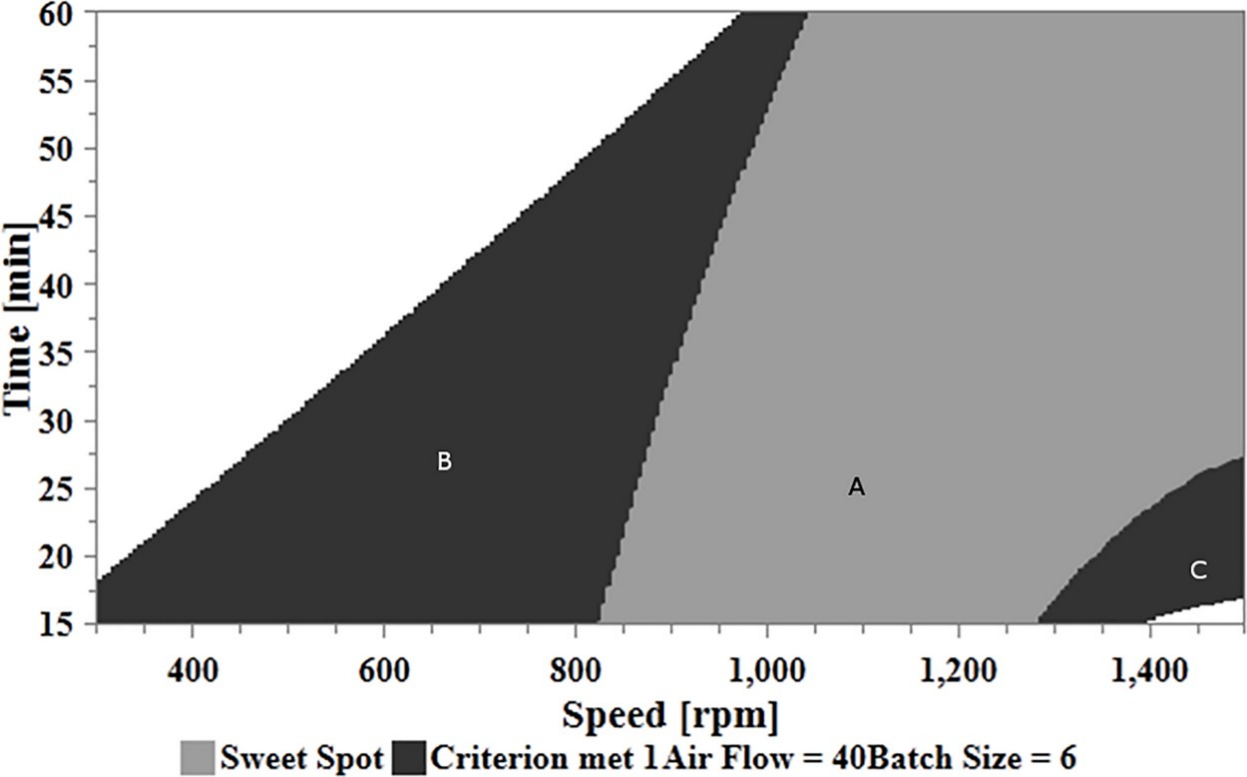
Sweet Spot for optimal ranges of the CPPs (speed and time) for the desired profile of CQAs (dissolution and content uniformity) while air flow and batch size were set at 40psi and 6g respectively.

Based on the results obtained from RSM plots throughout the study, the optimal operable zones within the CPPs that reveal the space that could result in optimal CQAs (dissolution rate of <85% and blend with RSD <5%), are illustrated in Fig 14. From the graph, the area (A) represents the design space with CPPs (speed and processing time) that could result in desired responses for both dissolution rate and content uniformity. The zones marked with (B) and (C) represent areas the CPPs (speed and processing time) meet the criteria for content uniformity alone, whereas, the white zone represents the CPPs when none of the criteria is met. The maximum operable space was obtained when the air pressure was set at its maximum range of 40 psi, the batch size was set at its lowest range of 6 g and the speed can be varied from 850 rpm to 1500 rpm with processing time ranging from 15 to 60 minutes.

## Conclusion

The application of QbD principles accompanied by the multifactorial design of experimental analysis enabled the proper understanding of process parameters, particularly those with direct impact on targeted outcomes or CQAs. The initial screening studies identified speed, air pressure, processing time and batch size as key process parameters; while content uniformity, dissolution rate, particle size and FTIR C = O intensity were identified as CQAs. These were employed for the optimisation study. Based on created models design space bordered by high pressure, low batch size, and a range of speeds and processing times was identified, and working within this space produced dry coated particles with targeted functionalities. In conclusion, the systematic application of QbD principles served two broad aims in this work. Firstly, to identify the influence of variations and interactions of the chosen factor on targeted quality attributes; and secondly to optimise the system to determine the factor combinations to yield the desired FP.

## Supporting information

S1 Data Design of experiments worksheet that operates with MODDE software. (MIP)
